# 2,2′-[1,1′-(Octane-1,8-diyldioxy­dinitrilo)diethyl­idyne]diphenol

**DOI:** 10.1107/S1600536809033959

**Published:** 2009-09-05

**Authors:** Wen-Kui Dong, Jun-Feng Tong, Jian Yao, Shang-Sheng Gong, Jian-Chao Wu

**Affiliations:** a School of Chemical and Biological Engineering, Lanzhou Jiaotong University, Lanzhou 730070, People’s Republic of China

## Abstract

The title compound, C_24_H_32_N_2_O_4_, has a crystallographic inversion centre at the mid-point of the central C—C bond. At each end of the mol­ecule, intra­molecular O—H⋯N hydrogen bonds generate six-membered *S*(6) ring motifs. The crystal structure is stabilized by pairs of weak inter­molecular C—H⋯O hydrogen bonds that link neighbouring mol­ecules into *R*_2_^2^(40) ring motifs, which in turn form infinite one-dimensional supra­molecular ribbon structures.

## Related literature

For background to oxime-based salen-type tetra­dentate ligands, see: Akine *et al.* (2005 ▶); Dong, He *et al.* (2009 ▶); Dong, Sun *et al.* (2009 ▶). For the synthesis, see: Dong *et al.* (2008 ▶). For related structures, see: Dong, Zhao *et al.* (2009 ▶); Etemadi *et al.* (2009 ▶). For information relating to C—H⋯O hydrogen bonds, see: Desiraju (1996 ▶). For graph-set notation, see: Bernstein *et al.* (1995 ▶).
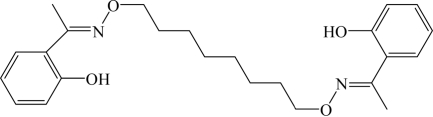


## Experimental

### 

#### Crystal data

C_24_H_32_N_2_O_4_
*M* *_r_* = 412.52
Monoclinic, 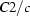

*a* = 12.9524 (12) Å
*b* = 4.6667 (6) Å
*c* = 37.722 (3) Å
β = 99.379 (2)°
*V* = 2249.6 (4) Å^3^
*Z* = 4
Mo *K*α radiation
μ = 0.08 mm^−1^
*T* = 298 K
0.50 × 0.48 × 0.20 mm

#### Data collection

Bruker SMART CCD area-detector diffractometer
Absorption correction: multi-scan (*SADABS*; Sheldrick, 1996 ▶) *T*_min_ = 0.960, *T*_max_ = 0.984
5371 measured reflections
1979 independent reflections
1172 reflections with *I* > 2σ(*I*)
*R*_int_ = 0.069

#### Refinement

*R*[*F* ^2^ > 2σ(*F* ^2^)] = 0.074
*wR*(*F* ^2^) = 0.173
*S* = 1.11
1979 reflections
137 parameters
H-atom parameters constrained
Δρ_max_ = 0.20 e Å^−3^
Δρ_min_ = −0.21 e Å^−3^

### 

Data collection: *SMART* (Siemens, 1996 ▶); cell refinement: *SAINT* (Siemens, 1996 ▶); data reduction: *SAINT*; program(s) used to solve structure: *SHELXS97* (Sheldrick, 2008 ▶); program(s) used to refine structure: *SHELXL97* (Sheldrick, 2008 ▶); molecular graphics: *SHELXTL* (Sheldrick, 2008 ▶) and *Mercury* (Macrae *et al.*, 2006 ▶); software used to prepare material for publication: *SHELXTL*.

## Supplementary Material

Crystal structure: contains datablocks global, I. DOI: 10.1107/S1600536809033959/pk2184sup1.cif

Structure factors: contains datablocks I. DOI: 10.1107/S1600536809033959/pk2184Isup2.hkl

Additional supplementary materials:  crystallographic information; 3D view; checkCIF report

## Figures and Tables

**Table 1:** Hydrogen-bond geometry (Å, °)

*D*—H⋯*A*	*D*—H	H⋯*A*	*D*⋯*A*	*D*—H⋯*A*
O2—H2⋯N1	0.82	1.84	2.558 (4)	145
C12—H12⋯O2^i^	0.93	2.64	3.544 (5)	164

Symmetry code: (i) 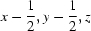.

**Fig. 1.**
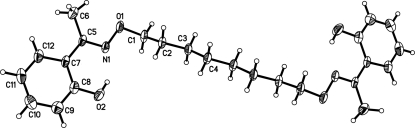
The molecular structure of the title compound with the atom numbering scheme. Unlabelled atoms are related to their labelled counterparts by the inversion operation [-x + 3/2,-y + 3/2,-z + 1]. Displacement ellipsoids for non-hydrogen atoms are drawn at the 30% probability level.

**Fig. 2.**
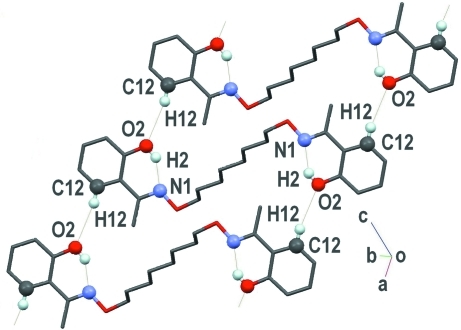
Part of the one-dimensional supramolecular structure of the title compound. Intramolecular and intermolecular hydrogen bonds are shown as dashed lines. Colour code: dark gray: C; red: O; blue: N; pale green: H (Macrae et al., 2006).

## supplementary crystallographic information

### Comment

Much attention has been focused on oxime-based salen-type tetradentate ligands in recent years due to their high stability against imine metathesis reactions (Akine *et al.*, 2005; Dong, He *et al.* 2009). A number of their metal complexes have been prepared and reported (Dong, Sun *et al.* 2009), which demonstrates that bisoxime ligands have strong coordinating ability with transition metals and non-transition metals.  In continuation of our previously reported works (Dong, Zhao *et al.* 2009), here we report synthesis and structure of salen-type bisoxime ligands, 2,2'-[1,1'-(octane-1,8-diyldioxydinitrilo)diethylidyne]diphenol.

The molecular structure of the title compound, as shown in Fig. 1, has a crystallographic inversion centre at the mid-point of the the central C—C bond.  Thus there is half a molecule in the asymmetric unit. The two benzene rings are parallel to each other with a perpendicular interplanar spacing of *ca* 5.316 (2) Å. In each molecule, there exist two intramolecular O—H···N hydrogen bonds, that form two S(6) ring motifs (Fig. 1) (Bernstein *et al.*, 1995). Pairs of weak intermolecular C—H···O hydrogen bonds (Desiraju, 1996) link neighbouring molecules into an infinite one-dimensional supramolecular structure with *R*_2_^2^(40) ring motifs (Table 1, Fig. 2), similar to that described by Etemadi *et al.*, (2009).

### Experimental

2,2'-[1,1'-(Octane-1,8-diyldioxydinitrilo)diethylidyne]diphenol was synthesized according to our previous work (Dong *et al.*, 2008). To an ethanol solution (4 ml) of 2'-hydroxyacetophenone (280.7 mg, 2.06 mmol) was added an ethanol solution (4 ml) of 1, 8-bis(aminooxy)octane (180.9 mg, 1.03 mmol). The mixture was stirred at 328–333 K for 48 h. When cooled to room temperature, the resulting white precipitate was filtered, and washed successively with ethanol and n-hexane. The product was dried under vacuum and purified by recrystallization from ethanol to yield 206.5 mg of the title compound. Yield, 49.01%. m. p. 345–347 K. Anal. Calcd. for C_24_H_32_N_2_O_4_: C, 69.88; H, 7.82; N, 6.79. Found: C, 69.50; H, 7.53; N, 6.87.

Colorless block-like single crystals suitable for X-ray diffraction studies were obtained after several days by slow evaporation from a diethyl ether solution.

### Refinement

Non-H atoms were refined anisotropically. H atoms were treated as riding atoms with distances C—H = 0.96 Å (CH_3_), 0.97 Å (CH_2_), 0.93 Å (CH), 0.82 Å (OH), and *U*_iso_(H) = 1.20 *U*_eq_(C) for methylene and methylidyne, 1.50 *U*_eq_(C) for methyl, 1.50 *U*_eq_(O).

### Crystal data

**Table:**

C_24_H_32_N_2_O_4_	*F*(000) = 888
*M**_r_* = 412.52	*D*_x_ = 1.218 Mg m^−^^3^
Monoclinic, *C*2/*c*	Melting point = 345–347 K
Hall symbol: -C 2yc	Mo *K*α radiation, λ = 0.71073 Å
*a* = 12.9524 (12) Å	Cell parameters from 1491 reflections
*b* = 4.6667 (6) Å	θ = 2.2–27.2°
*c* = 37.722 (3) Å	µ = 0.08 mm^−^^1^
β = 99.379 (2)°	*T* = 298 K
*V* = 2249.6 (4) Å^3^	Block-like, colorless
*Z* = 4	0.50 × 0.48 × 0.20 mm

### Data collection

**Table:**

Bruker SMART CCD area-detector diffractometer	1979 independent reflections
Radiation source: fine-focus sealed tube	1172 reflections with *I* > 2σ(*I*)
graphite	*R*_int_ = 0.069
φ and ω scans	θ_max_ = 25.0°, θ_min_ = 2.2°
Absorption correction: multi-scan (*SADABS*; Sheldrick, 1996)	*h* = −14→15
*T*_min_ = 0.960, *T*_max_ = 0.984	*k* = −5→5
5371 measured reflections	*l* = −37→44

### Refinement

**Table:**

Refinement on *F*^2^	Secondary atom site location: difference Fourier map
Least-squares matrix: full	Hydrogen site location: inferred from neighbouring sites
*R*[*F*^2^ > 2σ(*F*^2^)] = 0.074	H-atom parameters constrained
*wR*(*F*^2^) = 0.173	*w* = 1/[σ^2^(*F*_o_^2^) + (0.0225*P*)^2^ + 4.9486*P*] where *P* = (*F*_o_^2^ + 2*F*_c_^2^)/3
*S* = 1.11	(Δ/σ)_max_ < 0.001
1979 reflections	Δρ_max_ = 0.20 e Å^−^^3^
137 parameters	Δρ_min_ = −0.21 e Å^−^^3^
0 restraints	Extinction correction: *SHELXL97* (Sheldrick, 2008), Fc^*^=kFc[1+0.001xFc^2^λ^3^/sin(2θ)]^-1/4^
Primary atom site location: structure-invariant direct methods	Extinction coefficient: 0.0080 (9)

### Special details

**Table:**

Geometry. All e.s.d.'s (except the e.s.d. in the dihedral angle between two l.s. planes) are estimated using the full covariance matrix. The cell e.s.d.'s are taken into account individually in the estimation of e.s.d.'s in distances, angles and torsion angles; correlations between e.s.d.'s in cell parameters are only used when they are defined by crystal symmetry. An approximate (isotropic) treatment of cell e.s.d.'s is used for estimating e.s.d.'s involving l.s. planes.
Refinement. Refinement of *F*^2^ against ALL reflections. The weighted *R*-factor *wR* and goodness of fit *S* are based on *F*^2^, conventional *R*-factors *R* are based on *F*, with *F* set to zero for negative *F*^2^. The threshold expression of *F*^2^ > σ(*F*^2^) is used only for calculating *R*-factors(gt) *etc*. and is not relevant to the choice of reflections for refinement. *R*-factors based on *F*^2^ are statistically about twice as large as those based on *F*, and *R*- factors based on ALL data will be even larger.

### Fractional atomic coordinates and isotropic or equivalent isotropic displacement parameters (Å^2^)

**Table:**

	*x*	*y*	*z*	*U*_iso_*/*U*_eq_	
N1	0.3869 (2)	0.5102 (7)	0.38238 (7)	0.0490 (8)	
O1	0.35769 (17)	0.6700 (6)	0.41082 (6)	0.0617 (8)	
O2	0.51778 (18)	0.3352 (7)	0.34323 (7)	0.0746 (9)	
H2	0.4980	0.4288	0.3593	0.112*	
C1	0.4459 (3)	0.8346 (9)	0.42728 (9)	0.0570 (10)	
H1A	0.4224	0.9786	0.4427	0.068*	
H1B	0.4757	0.9335	0.4087	0.068*	
C2	0.5297 (2)	0.6538 (9)	0.44922 (9)	0.0513 (9)	
H2A	0.5594	0.5254	0.4333	0.062*	
H2B	0.4983	0.5377	0.4659	0.062*	
C3	0.6162 (2)	0.8345 (9)	0.46995 (9)	0.0518 (9)	
H3A	0.5870	0.9513	0.4872	0.062*	
H3B	0.6430	0.9627	0.4534	0.062*	
C4	0.7063 (2)	0.6595 (9)	0.48983 (9)	0.0540 (10)	
H4A	0.6796	0.5333	0.5067	0.065*	
H4B	0.7347	0.5406	0.4727	0.065*	
C5	0.3132 (2)	0.3491 (8)	0.36639 (8)	0.0440 (9)	
C6	0.2085 (3)	0.3295 (12)	0.37779 (11)	0.0786 (14)	
H6A	0.1970	0.1371	0.3852	0.118*	
H6B	0.1552	0.3805	0.3580	0.118*	
H6C	0.2059	0.4585	0.3975	0.118*	
C7	0.3382 (2)	0.1742 (8)	0.33664 (8)	0.0447 (9)	
C8	0.4372 (3)	0.1723 (9)	0.32643 (10)	0.0547 (10)	
C9	0.4582 (3)	0.0008 (10)	0.29864 (11)	0.0699 (12)	
H9	0.5247	0.0013	0.2924	0.084*	
C10	0.3815 (4)	−0.1708 (10)	0.28013 (10)	0.0714 (12)	
H10	0.3961	−0.2862	0.2615	0.086*	
C11	0.2835 (4)	−0.1710 (10)	0.28930 (10)	0.0699 (12)	
H11	0.2313	−0.2859	0.2767	0.084*	
C12	0.2619 (3)	−0.0026 (9)	0.31692 (9)	0.0587 (11)	
H12	0.1948	−0.0054	0.3228	0.070*	

### Atomic displacement parameters (Å^2^)

**Table:**

	*U*^11^	*U*^22^	*U*^33^	*U*^12^	*U*^13^	*U*^23^
N1	0.0372 (16)	0.058 (2)	0.0459 (16)	0.0005 (15)	−0.0117 (12)	−0.0031 (17)
O1	0.0381 (14)	0.081 (2)	0.0591 (15)	0.0022 (14)	−0.0132 (11)	−0.0207 (16)
O2	0.0399 (14)	0.092 (2)	0.0886 (19)	−0.0111 (16)	0.0020 (13)	−0.0267 (19)
C1	0.044 (2)	0.061 (2)	0.057 (2)	0.001 (2)	−0.0190 (16)	−0.017 (2)
C2	0.0416 (19)	0.060 (2)	0.0460 (19)	−0.0056 (19)	−0.0126 (15)	−0.001 (2)
C3	0.0413 (19)	0.060 (2)	0.0483 (19)	−0.002 (2)	−0.0097 (15)	−0.012 (2)
C4	0.0395 (19)	0.061 (2)	0.056 (2)	−0.004 (2)	−0.0099 (15)	−0.009 (2)
C5	0.0320 (18)	0.050 (2)	0.0437 (18)	−0.0007 (17)	−0.0128 (14)	0.0062 (19)
C6	0.045 (2)	0.108 (4)	0.080 (3)	−0.017 (3)	0.0029 (19)	−0.024 (3)
C7	0.0380 (19)	0.046 (2)	0.0433 (18)	−0.0042 (17)	−0.0124 (14)	0.0072 (18)
C8	0.050 (2)	0.054 (2)	0.055 (2)	−0.001 (2)	−0.0078 (17)	−0.003 (2)
C9	0.061 (3)	0.080 (3)	0.067 (3)	0.002 (3)	0.005 (2)	−0.003 (3)
C10	0.092 (3)	0.068 (3)	0.051 (2)	0.005 (3)	0.001 (2)	−0.002 (2)
C11	0.083 (3)	0.066 (3)	0.051 (2)	−0.019 (3)	−0.016 (2)	0.003 (2)
C12	0.055 (2)	0.064 (3)	0.051 (2)	−0.012 (2)	−0.0112 (17)	0.009 (2)

### Geometric parameters (Å, °)

**Table:**

N1—C5	1.285 (4)	C4—H4B	0.9700
N1—O1	1.408 (3)	C5—C7	1.466 (5)
O1—C1	1.432 (4)	C5—C6	1.491 (5)
O2—C8	1.361 (4)	C6—H6A	0.9600
O2—H2	0.8200	C6—H6B	0.9600
C1—C2	1.511 (5)	C6—H6C	0.9600
C1—H1A	0.9700	C7—C8	1.398 (5)
C1—H1B	0.9700	C7—C12	1.404 (5)
C2—C3	1.514 (5)	C8—C9	1.380 (5)
C2—H2A	0.9700	C9—C10	1.375 (6)
C2—H2B	0.9700	C9—H9	0.9300
C3—C4	1.518 (5)	C10—C11	1.369 (5)
C3—H3A	0.9700	C10—H10	0.9300
C3—H3B	0.9700	C11—C12	1.370 (5)
C4—C4^i^	1.517 (6)	C11—H11	0.9300
C4—H4A	0.9700	C12—H12	0.9300
			
C5—N1—O1	113.3 (3)	N1—C5—C7	116.5 (3)
N1—O1—C1	108.7 (3)	N1—C5—C6	122.8 (3)
C8—O2—H2	109.5	C7—C5—C6	120.7 (3)
O1—C1—C2	112.9 (3)	C5—C6—H6A	109.5
O1—C1—H1A	109.0	C5—C6—H6B	109.5
C2—C1—H1A	109.0	H6A—C6—H6B	109.5
O1—C1—H1B	109.0	C5—C6—H6C	109.5
C2—C1—H1B	109.0	H6A—C6—H6C	109.5
H1A—C1—H1B	107.8	H6B—C6—H6C	109.5
C1—C2—C3	112.1 (3)	C8—C7—C12	116.7 (3)
C1—C2—H2A	109.2	C8—C7—C5	122.7 (3)
C3—C2—H2A	109.2	C12—C7—C5	120.6 (3)
C1—C2—H2B	109.2	O2—C8—C9	116.5 (4)
C3—C2—H2B	109.2	O2—C8—C7	122.4 (3)
H2A—C2—H2B	107.9	C9—C8—C7	121.0 (4)
C2—C3—C4	113.6 (3)	C10—C9—C8	120.5 (4)
C2—C3—H3A	108.8	C10—C9—H9	119.7
C4—C3—H3A	108.8	C8—C9—H9	119.7
C2—C3—H3B	108.8	C11—C10—C9	119.7 (4)
C4—C3—H3B	108.8	C11—C10—H10	120.2
H3A—C3—H3B	107.7	C9—C10—H10	120.2
C4^i^—C4—C3	113.6 (4)	C10—C11—C12	120.3 (4)
C4^i^—C4—H4A	108.8	C10—C11—H11	119.9
C3—C4—H4A	108.8	C12—C11—H11	119.9
C4^i^—C4—H4B	108.8	C11—C12—C7	121.8 (4)
C3—C4—H4B	108.8	C11—C12—H12	119.1
H4A—C4—H4B	107.7	C7—C12—H12	119.1
			
C5—N1—O1—C1	178.4 (3)	C12—C7—C8—O2	179.6 (3)
N1—O1—C1—C2	−72.6 (4)	C5—C7—C8—O2	−0.6 (6)
O1—C1—C2—C3	−173.2 (3)	C12—C7—C8—C9	−1.0 (5)
C1—C2—C3—C4	−175.1 (3)	C5—C7—C8—C9	178.8 (3)
C2—C3—C4—C4^i^	179.2 (4)	O2—C8—C9—C10	−180.0 (4)
O1—N1—C5—C7	−179.5 (3)	C7—C8—C9—C10	0.6 (6)
O1—N1—C5—C6	−1.6 (5)	C8—C9—C10—C11	0.1 (6)
N1—C5—C7—C8	2.0 (5)	C9—C10—C11—C12	−0.4 (6)
C6—C5—C7—C8	−176.0 (4)	C10—C11—C12—C7	0.0 (6)
N1—C5—C7—C12	−178.2 (3)	C8—C7—C12—C11	0.7 (5)
C6—C5—C7—C12	3.8 (5)	C5—C7—C12—C11	−179.1 (4)

Symmetry codes: (i) −*x*+3/2, −*y*+3/2, −*z*+1.

### Hydrogen-bond geometry (Å, °)

**Table:**

*D*—H···*A*	*D*—H	H···*A*	*D*···*A*	*D*—H···*A*
O2—H2···N1	0.82	1.84	2.558 (4)	145
C12—H12···O2^ii^	0.93	2.64	3.544 (5)	164

Symmetry codes: (ii) *x*−1/2, *y*−1/2, *z*.

## References

[bb1] Akine, S., Taniguchi, T., Dong, W. K., Masubuchi, S. & Nabeshima, T. (2005). *J. Org. Chem.* **70**, 1704–1711. 10.1021/jo048030y 15730291

[bb2] Bernstein, J., Davis, R. E., Shimoni, L. & Chang, N.-L. (1995). *Angew. Chem. Int. Ed. Engl.* **34**, 1555–1573.

[bb3] Desiraju, G. R. (1996). *Acc. Chem. Res.* **29**, 441–449. 10.1021/ar950135n 23618410

[bb4] Dong, W. K., He, X. N., Yan, H. B., Lv, Z. W., Chen, X. W. K., Zhao, C. Y. & Tang, X. L. (2009). *Polyhedron*, **28**, 1419–1428.

[bb5] Dong, W.-K., He, X.-N., Zhong, J.-K., Chen, X. & Yu, T.-Z. (2008). *Acta Cryst.* E**64**, o1098. 10.1107/S1600536808012701 PMC2961531 21202612

[bb6] Dong, W. K., Sun, Y. X., Zhang, Y. P., Li, L., He, X. N. & Tang, X. L. (2009). *Inorg. Chim. Acta*, **362**, 117–124.

[bb7] Dong, W. K., Zhao, C. Y., Sun, Y. X., Tang, X. L. & He, X. N. (2009). *Inorg. Chem. Commun.* **12**, 234–236.

[bb8] Etemadi, B., Kia, R., Sharghi, H. & Hosseini Sarvari, M. (2009). *Acta Cryst.* E**65**, o1309. 10.1107/S1600536809016663 PMC2969837 21583166

[bb9] Macrae, C. F., Edgington, P. R., McCabe, P., Pidcock, E., Shields, G. P., Taylor, R., Towler, M. & van de Streek, J. (2006). *J. Appl. Cryst.* **39**, 453–457.

[bb10] Sheldrick, G. M. (1996). *SADABS* University of Göttingen, Germany.

[bb11] Sheldrick, G. M. (2008). *Acta Cryst.* A**64**, 112–122. 10.1107/S0108767307043930 18156677

[bb12] Siemens (1996). *SMART* and *SAINT* Siemens Analytical X-ray Instruments Inc., Madison, Wisconsin, USA.

